# Expedition cruises, island hopping, and zoonotic risk: governance and operational lessons from the MV *Hondius* Andes hantavirus outbreak

**DOI:** 10.3389/fmed.2026.1892006

**Published:** 2026-07-16

**Authors:** Esteban Ortiz-Prado, María Paz Cadena, María A. Vethencourt-Ysea, Jorge Vasconez-Gonzalez, Kirk O. Douglas, Juan S. Izquierdo-Condoy

**Affiliations:** 1 One Health Research Group, Universidad de Las Americas, Quito, Ecuador; 2 Universidad Latina de Costa Rica, San José, Costa Rica; 3 Centre for Biosecurity Studies, The University of the West Indies, St. Michael, Barbados

**Keywords:** Andes virus, ecological tourism, expedition cruises, One Health, orthohantavirus, travel-associated infections

## Abstract

The Andes orthohantavirus outbreak linked to the MV *Hondius* expedition cruise illustrates how a probable land-based zoonotic exposure can become a multinational public health event when it intersects with enclosed shipboard environments, delayed clinical recognition, remote navigation, medical evacuation, and international passenger dispersal. Although hantavirus infection is classically associated with exposure to infected rodents or contaminated environments, Andes virus is exceptional among orthohantaviruses because limited person-to-person transmission has been documented, particularly after close and prolonged contact. This Perspective uses the MV *Hondius* outbreak as an analytical case study to identify governance and operational gaps in expedition-era travel medicine. Existing International Health Regulations, WHO ship-event guidance, and ECDC recommendations provide essential foundations for coordination, notification, isolation, and contact tracing; however, this outbreak exposed expedition-specific gaps in safe port access, medical evacuation, onboard recognition of nonspecific febrile illness, diagnostic escalation, passenger traceability, and post-disembarkation monitoring. We therefore propose an accountability-oriented One Health preparedness model that operationalizes existing guidance through route-level risk assessment, exposure-history assessment, onboard syndromic surveillance, isolation and telemedicine triggers, reference-laboratory pathways, port-of-call agreements, auditable passenger and excursion records, and cross-border post-travel monitoring. The central lesson is not that expedition cruises, birdwatching, or ecological tourism are inherently unsafe, but that their expanding geographic reach requires binding, auditable, and expedition-specific outbreak protocols before passengers embark.

## 1 Introduction

The recent Andes orthohantavirus outbreak linked to the MV *Hondius* expedition cruise should be interpreted as more than an isolated maritime health event. It represents a rare and highly instructive One Health case study in which ecological exposure, international tourism, wildlife-associated environments, enclosed transport systems, delayed diagnosis, intensive care needs, and multi-country public health coordination converged within a single epidemiological chain. The event illustrates how a pathogen usually embedded in regional rodent–environment interfaces can become internationally disruptive when introduced into high-mobility travel networks.

On 2 May 2026, the World Health Organization (WHO) was notified of a cluster of severe acute respiratory illness aboard the Dutch-flagged expedition cruise ship MV *Hondius* (1). By 25 May 2026, the outbreak linked to the MV *Hondius* had increased to 13 reported cases and three deaths, corresponding to an observed case fatality ratio of 25% (2). No additional deaths had been reported since 2 May, but more than 600 contacts across 30 countries remained under public health monitoring. Spain reported the most recent additional case in a traveler who was evacuated from the cruise and is isolating at a hospital in Madrid for hantavirus (3). Earlier official reports had documented six laboratory-confirmed Andes virus infections by PCR or sequencing and two probable cases; subsequent updates expanded the number of reported cases as additional infections were identified among repatriated passengers and crew. The vessel had departed Ushuaia, Argentina, on 1 April 2026, after several passengers had travelled through areas of Argentina, Chile, and Uruguay before boarding (2). The initial WHO report described the probable index case as an adult male who had travelled through the Southern Cone for more than 3 months before embarkation, developed symptoms on 6 April, and died onboard on 11 April. His close contact and travel companion later deteriorated after disembarkation and died in Johannesburg on 26 April; she was subsequently confirmed by PCR as infected with hantavirus. Subsequent clinical and genomic investigations confirmed Andes virus infection in additional cases, with early sequencing closely matching previously reported Argentine sequences, supporting a likely zoonotic introduction before departure while leaving open the contribution of limited person-to-person transmission aboard the vessel (1). This distinction is important because Andes virus is the only orthohantavirus with documented person-to-person transmission, typically after close and prolonged contact (1, 4).

Orthohantaviruses are zoonotic viruses maintained primarily in rodent reservoirs (5). Human infection usually occurs through inhalation of aerosolized particles from rodent urine, feces, or saliva, particularly in contaminated enclosed spaces or heavily infested environments (6). Clinically, orthohantavirus infections are classically associated with two major syndromic patterns: hemorrhagic fever with renal syndrome, mainly reported in Europe and Asia, and hantavirus cardiopulmonary syndrome, predominantly described in the Americas; nephropathia epidemica is generally considered a milder form of hemorrhagic fever with renal syndrome (7, 8). In the Americas, orthohantavirus infection can lead to hantavirus cardiopulmonary syndrome, a severe illness characterized by an initial febrile or gastrointestinal phase followed, in some cases, by rapid cardiopulmonary deterioration (9, 10).

This Perspective uses the MV *Hondius* outbreak as an analytical case study to identify governance and operational gaps in outbreak preparedness for expedition cruises. Rather than proposing a framework that duplicates existing IHR 2005, WHO, or ECDC mechanisms, we ask whether these mechanisms are sufficiently operational for remote, high-mobility, expedition-specific contexts. The outbreak exposed two critical vulnerabilities: the absence of binding, pre-arranged protocols for safe port access and medical evacuation of vessels with suspected or confirmed high-consequence infections, and the lack of a standardized passenger traceability system capable of supporting rapid post-disembarkation monitoring across multiple jurisdictions. The central lesson is therefore not only that expedition cruises require One Health awareness, but that this awareness must be translated into enforceable, accountable, and auditable preparedness arrangements before departure.

## 2 Cruise ships as mobile mass-gathering platforms: enclosed, crowded, isolated and outbreak-prone

Cruise ships are not merely transport platforms, they are temporary mobile communities involving passengers and crew who share cabins, dining areas, corridors, recreational spaces, medical facilities, ventilation systems, sanitation infrastructure, and disembarkation pathways (11, 12). Expedition cruises add further complexity as they deliberately move passengers into remote, wildlife diverse, and often under-surveilled environments where human–animal–environment interfaces are intense and public health infrastructure may be limited (13, 14).

From an epidemiological perspective, cruise ships can combine several outbreak-promoting conditions. First, high-density human interaction in semi-enclosed spaces is facilitated. Second, a significant proportion of older passengers, many of whom may have comorbidities, increasing the risk of severe outcomes after infection are repeat cruise passengers (15). Third, their operations are often far from tertiary hospitals, making diagnosis, isolation, evacuation, and access to intensive care logistically difficult (13). Fourth, they connect multiple jurisdictions within short periods, complicating case investigation, contact tracing, quarantine decisions, and risk communication (15, 16) (Table 1).

**Table 1:** The factors, epidemiological relevance and One Health implication of how expedition cruises can amplify infectious disease risk.

Cruise-related factor	Epidemiological relevance	One Health implication
High passenger density	Frequent close contact in shared indoor spaces	Increased risk of transmission for pathogens capable of human-to-human spread
Enclosed environments	Cabins, dining rooms, lecture halls, corridors, and medical rooms may concentrate exposure	Need for ventilation, isolation protocols, and infection prevention measures
Older passenger demographics	Many expedition cruises attract older adults, sometimes with comorbidities	Higher risk of severe disease, hospitalization, and intensive care need
Remote navigation	Ships may be days away from tertiary hospitals	Requires pre-planned evacuation and telemedicine pathways
Multi-country itineraries	Passengers may disembark in several jurisdictions	Requires international contact tracing and IHR coordination
Delayed symptom recognition	Early hantavirus symptoms may resemble influenza-like or gastrointestinal illness	Need for shipboard syndromic surveillance
Long incubation period	Andes virus symptoms may appear weeks after exposure	Requires post-travel monitoring and risk-stratified follow-up
Limited onboard diagnostics	Ship medical units usually lack advanced pathogen testing	Need for rapid molecular platforms and reference-laboratory networks

**Figure 1:**
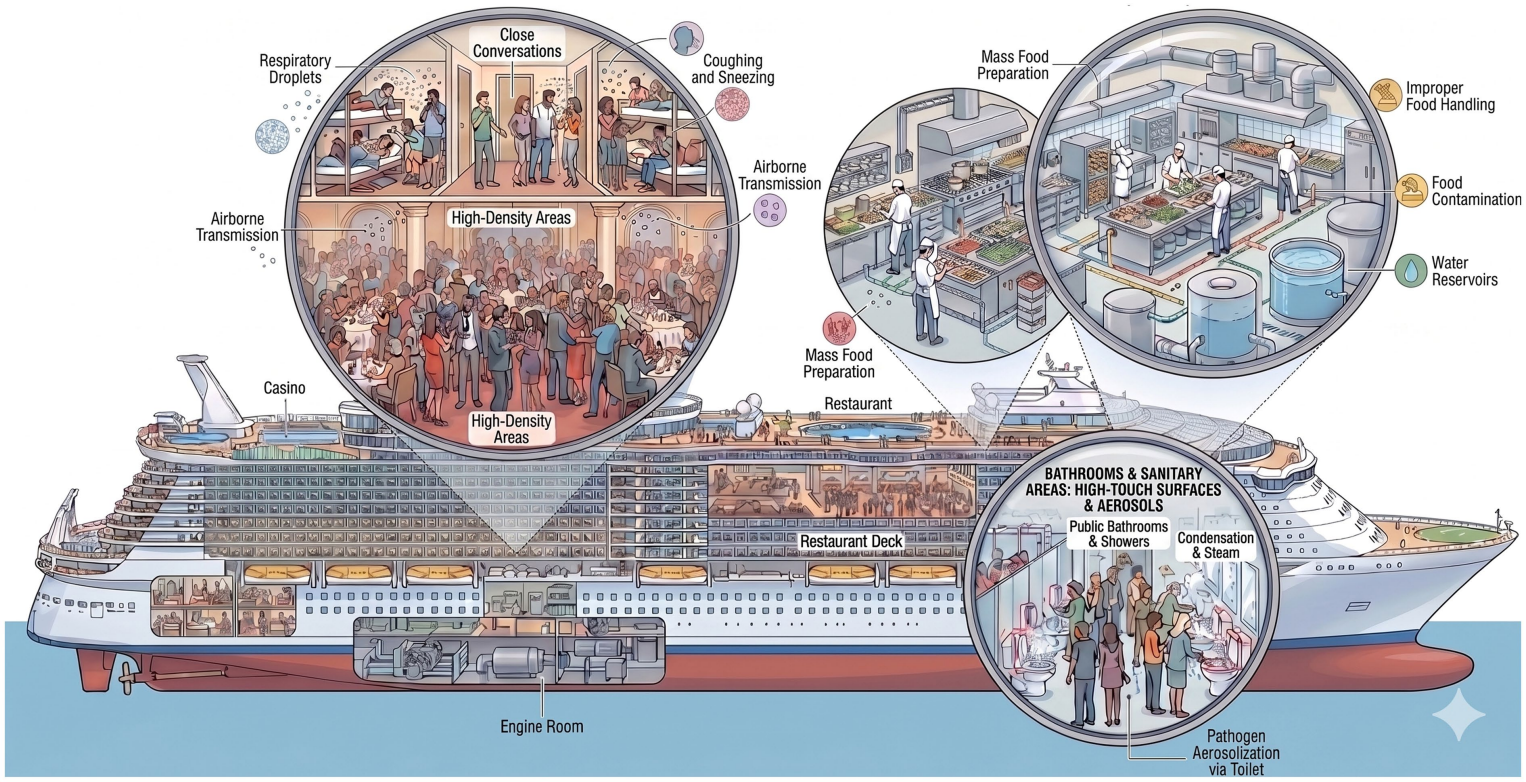
Infectious disease transmission pathways on cruise ships. Schematic cutaway illustration of a cruise ship showing key environments that can amplify infectious disease transmission, including passenger cabins, entertainment areas, shared dining spaces, galleys and food-preparation zones, water reservoirs, bathrooms, and sanitary areas. Magnified callouts highlight major transmission mechanisms, including respiratory droplets, airborne spread, close-contact interactions, foodborne contamination, high-touch surfaces, and toilet-associated aerosolization.

Finally, due to passenger disembarkment at ports and subsequent repatriation through commercial or charter flights, a shipboard event can rapidly become a multi-country public health challenge. The MV *Hondius* event illustrates some of these population’s dynamics. The outbreak involved passengers and crew from multiple countries, required medical evacuation, laboratory testing across international networks, and coordination through the International Health Regulations. The long incubation period of Andes virus infection further complicated public health management, because exposed individuals required monitoring for up to 42 days after their last potential exposure (17) (Figure 1).

Importantly, the MV *Hondius* event should not be interpreted as evidence that the cruise ship itself was the ecological source of infection. Orthohantaviruses are primarily maintained in rodent reservoirs, and human infection usually follows environmental exposure to contaminated rodent excreta. In this case, the clinical timeline suggests that the probable index case was likely infected before boarding, most plausibly during travel in the Southern Cone of South America, with symptom onset occurring only after the ship had departed (2). This distinction is essential: the central epidemiological question concerns the pre-boarding exposure history of the index case, particularly during the weeks before embarkation. Nevertheless, once illness developed onboard, the cruise ship became a public health amplifier of complexity. Close quarters, shared spaces, delayed clinical recognition, limited onboard diagnostic capacity, and international passenger movement transformed a likely land-based zoonotic exposure into a multinational surveillance, communication, and response challenge. Thus, the risk posed by cruise ships in such events lies not in serving as the natural reservoir, but in their capacity to complicate detection, containment, contact tracing, and cross-border coordination.

This epidemiological context also defines the limits of pre-boarding screening. If the probable index case acquired infection before embarkation and was still within the incubation period at the time of boarding, symptom-based screening alone would not have detected the risk. Therefore, the main operational lesson is not that pre-boarding screening should be treated as the primary safeguard, but that expedition cruises require layered detection and escalation systems after departure. These include structured exposure-history assessment, early onboard recognition of nonspecific febrile or gastrointestinal illness, immediate isolation triggers, differential diagnosis algorithms for severe travel-associated infections, telemedicine consultation, and pre-arranged diagnostic referral pathways. For long-incubation zoonoses, preparedness must extend beyond embarkation and continue throughout the voyage and after disembarkation.

## 3 The global scale of cruise tourism

**Figure 2:**
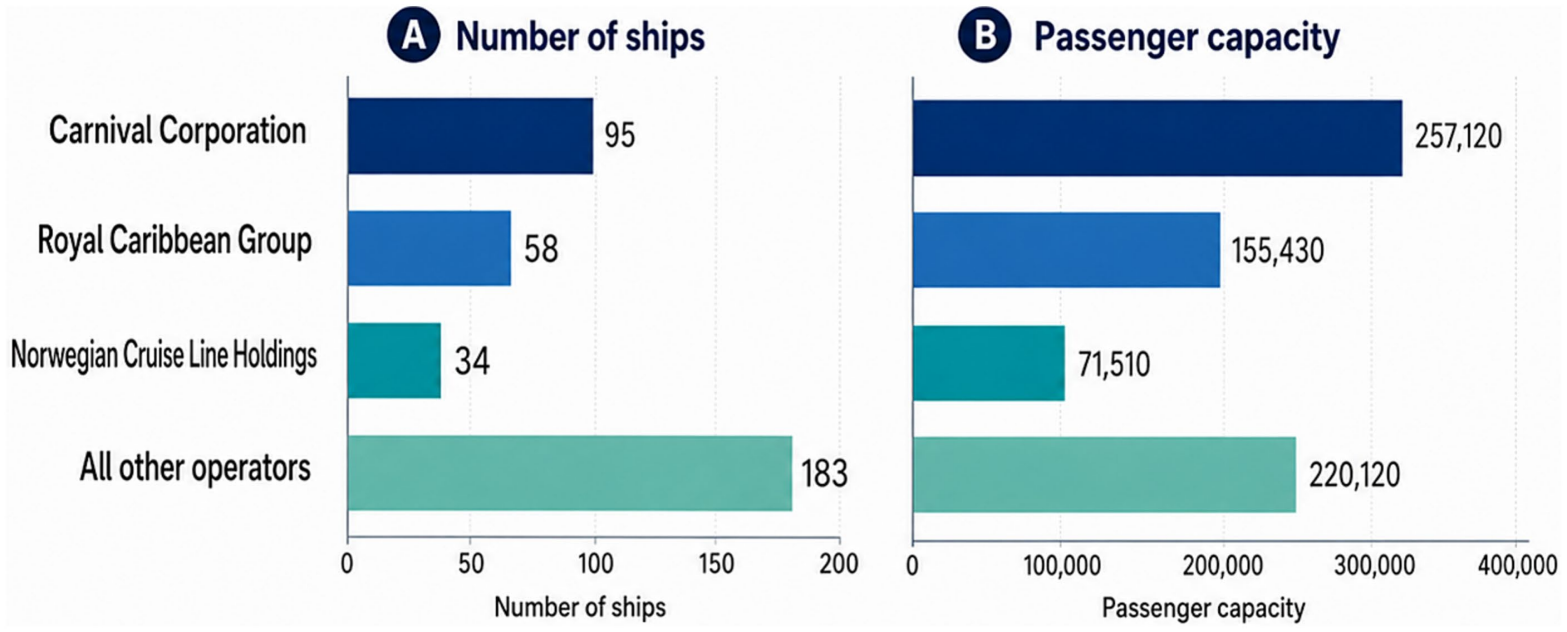
Global ocean cruise industry capacity by major operator groups, end of 2025. Expedition and polar cruise vessels, including operators such as Hurtigruten, Lindblad Expeditions, Quark Expeditions, Ponant, and Oceanwide Expeditions, represent a growing niche within the “all other operators” category and frequently operate island-hopping routes in remote ecosystems. Source: Cruise Market Watch, 2025.

The global cruise industry has reached an unprecedented scale, creating important One Health challenges for zoonotic disease emergence, detection, and dissemination. By the end of 2025, the worldwide ocean cruise fleet was estimated at approximately 370 ships, with a total passenger capacity of about 704,200 berths (18). Major operators dominate this capacity: Carnival Corporation accounted for approximately 95 ships and 257,120 berths; Royal Caribbean Group for 58 ships and 155,430 berths; and Norwegian Cruise Line Holdings, including Oceania Cruises and Regent Seven Seas Cruises, for 34 ships and 71,510 berths. The remaining 183 vessels, including luxury, niche, and expedition operators, represented approximately 220,120 berths (Figure 2).

This scale is epidemiologically relevant. The Cruise Lines International Association reported that global ocean cruise passenger volume reached a historic high of 37. million passengers in 2025 (19), while independent industry estimates projected more than 38 million passengers in 2026 (20). Although mass-market vessels account for most global capacity, the expedition cruise segment including small, specialized ships such as the MV *Hondius*, with a capacity of approximately 170–196 passengers is expanding in response to demand for immersive nature-based travel. These vessels routinely perform island-hopping itineraries through remote and ecologically sensitive regions, including Patagonia, Antarctica, South Georgia, the South Atlantic islands, and other biodiversity-rich destinations. With continued fleet growth and diversification of cruise itineraries, the frequency of human–animal–environment interfaces in zoonotic hotspots is likely to increase, amplifying the public health implications illustrated by the MV *Hondius* Andes virus outbreak.

## 4 Ecological tourism, island hopping and overlooked exposure interfaces

**Figure 3:**
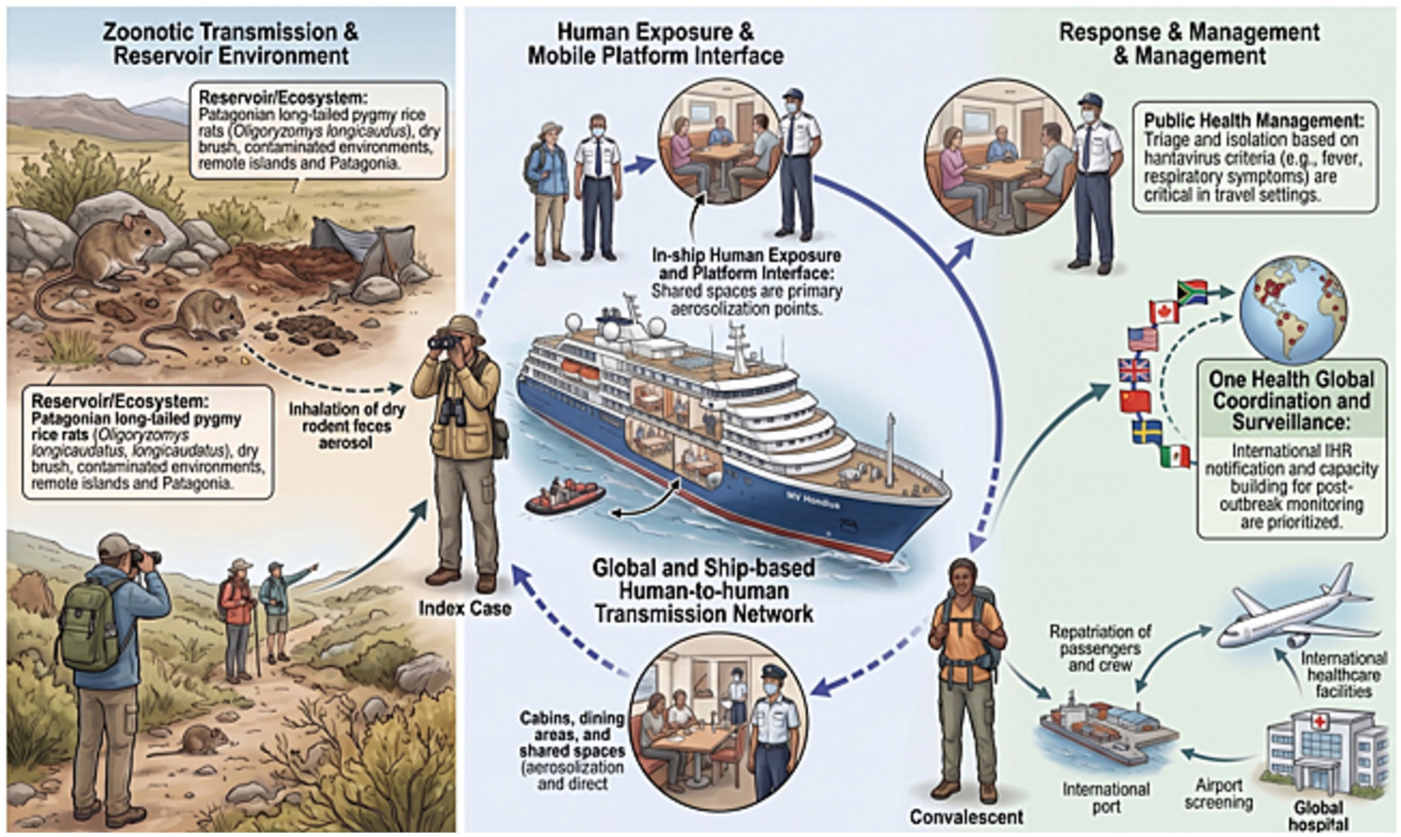
One Health transmission pathway and international response framework for a suspected cruise ship–associated zoonotic outbreak. The MV *Hondius* outbreak illustrates the need for anticipatory One Health surveillance across ecological and travel corridors rather than reactive management after international dispersion has occurred. This figure illustrates the proposed One Health transmission and response interface for hantavirus risk associated with expedition cruises. The diagram integrates five interconnected domains: (1) the reservoir and ecosystem interface, including rodents, contaminated environments, remote islands, and Patagonia; (2) human exposure, involving tourists, birdwatchers, guides, crew, and local communities; (3) the mobile platform, represented by the expedition cruise, cabins, dining areas, and shared indoor spaces; (4) international dispersion through ports, flights, repatriation, and hospitals; and (5) the public health response, including diagnostics, isolation, International Health Regulations coordination, sequencing, surveillance, and contact tracing.

Ecological tourism is often framed as low-impact because it is educational, conservation-oriented, or scientifically motivated (21). However, the MV *Hondius* outbreak underscores that some forms of ecotourism can place travelers in contact with underrecognized zoonotic interfaces. Birdwatchers, wildlife photographers, naturalists, expedition guides, and adventure tourists may visit wetlands, forest edges, rural roads, islands, docks, farms, abandoned structures, storage areas, rustic cabins, food markets, landfills, and waste-disposal sites (22–24). These settings are transition zones where humans, wildlife, synanthropic animals, rodents, food waste, contaminated dust, and environmental pathogens may converge (Figure 3).

In the current Andes hantavirus cruise outbreak, the probable index case was described in official reports as an older adult traveler who had spent several months in the Southern Cone before embarkation and developed symptoms after departure (1). The individual is not named here because personal identification does not add to the epidemiological assessment of the outbreak and is not necessary for public health interpretation. Although media reports have discussed possible exposure sites, no definitive source of infection has been confirmed. Therefore, this outbreak should be interpreted cautiously. Its value lies in illustrating how expedition cruises and ecological tourism may create opportunities for zoonotic exposure and subsequent outbreak amplification in enclosed travel settings, but the available evidence does not confirm a specific source, site of exposure, or transmission pathway. The landfill hypothesis should not be presented as a confirmed source of infection; rather, it is best understood as an example of an overlooked One Health interface where birds, rodents, waste, tourists, domestic animals, and local environments may intersect.

Beyond landfills, expedition travel may involve multiple additional exposure points.

Dogs for instance, and other domestic animals may act as sentinels or mechanical bridges between human spaces and wildlife-associated environments, even though they are not considered primary reservoirs for orthohantaviruses. Street food areas, local markets, poorly stored food, docks, camping sites, field stations, rustic lodges, and storage rooms may attract rodents or other animals (25, 26). In poorly ventilated or rodent-infested structures, contaminated dust containing rodent excreta may become aerosolized during cleaning, movement, or disturbance (27). Visits to islands may also involve repeated landings, short exposure windows, rapid movement between ecological niches, and limited environmental health assessment (28, 29). The result is a travel pattern in which tourists move from one ecological interface to another, often faster than public health systems can reconstruct or evaluate exposure risk.

This interpretation does not imply that tourists, ornithologists, expedition guides, or cruise operators should be blamed. Instead, it suggests that modern ecological tourism has outpaced the public health frameworks designed to monitor it. Tourists are not passive recipients of risk; they are mobile participants in a shared One Health system. A traveler may be exposed in one ecological setting, become symptomatic in another jurisdiction, receive care in a third country, and require monitoring across several national health systems. Table 2 summarizes potential exposure interfaces, plausible risk mechanisms, and examples of prevention or surveillance measures that could reduce the risk of zoonotic pathogen transmission during expedition travel.

**Table 2:** Tourist and expedition-related exposure interfaces relevant to One Health surveillance.

Exposure interface	Possible risk mechanism	Examples of prevention or monitoring
Landfills and waste-disposal areas	Food waste may attract birds, rodents, dogs, and other synanthropic animals	Avoid informal access; conduct environmental risk mapping; strengthen rodent surveillance
Birdwatching sites	Travelers may enter wetlands, forest edges, rural roads, or waste-adjacent areas	Provide pre-travel ecological risk briefings; maintain safe distance from animal congregation sites
Rustic cabins or field stations	Poor ventilation and rodent contamination may increase inhalation risk	Inspect before use; ventilate enclosed spaces; avoid sweeping dry dust; use PPE when cleaning
Docks and storage areas	Food storage, cargo, and waste may attract rodents	Implement rodent control; seal food storage; conduct routine inspections
Street food areas and open markets	Food waste may attract rodents, dogs, and other animals	Reinforce food hygiene guidance; improve waste management; avoid visibly contaminated environments
Remote islands	Limited surveillance and repeated landings may obscure exposure timing	Conduct landing-site risk assessments; maintain activity logs; implement post-landing symptom surveillance
Dogs and domestic animals	Domestic animals may indicate environmental contamination or move between human and wildlife spaces	Treat them as sentinels of environmental hygiene, not as proven orthohantavirus reservoirs
Hiking and photography sites	Close contact with soil, dust, vegetation, burrows, or animal nesting areas may increase exposure	Avoid disturbing rodent habitats; promote hand hygiene; use protective footwear
Cruise excursions	Short, repeated exposures across multiple ecological sites may complicate retrospective exposure reconstruction	Maintain detailed excursion and activity logs for contact tracing and exposure assessment

PPE, Personal protective equipment.

## 5 Cruise medical units and diagnostic preparedness: from basic care to mobile molecular surveillance

The MV *Hondius* outbreak also raises an important operational question: what diagnostic capacity should expedition cruises carry when travelling through remote, zoonosis-prone regions? Traditional ship medical units are designed primarily for acute stabilization, trauma, common infections, motion sickness, cardiovascular events, and emergency evacuation. There is often a lack capacity to diagnose rare zoonotic infections in real time. However, expedition cruises increasingly enter remote ecological corridors where delayed diagnosis may have international public health consequences.

In orthohantavirus infection, early symptoms are nonspecific and may include fever, chills, myalgia, headache, gastrointestinal symptoms, malaise, and, in severe cases, later respiratory distress (30). These manifestations can overlap with influenza, COVID-19, bacterial or atypical pneumonia, gastroenteritis, dengue, leptospirosis, rickettsioses, and other travel-associated infections (31, 32). Without diagnostic support, the ship physician may face a broad differential diagnosis with limited capacity for laboratory confirmation. In the MV *Hondius* event, confirmation required PCR, sequencing, medical evacuation, and international laboratory collaboration (1). Due to operational constraints, infrastructure limitations, the lack of specific laboratory materials and equipment, and the absence of personnel trained in molecular testing, it is not feasible to perform these tests on board. Instead, efforts should focus on the development and implementation of standardized protocols for sample collection and preservation, maintaining appropriate cold-chain transport conditions, and ensuring effective coordination with public health authorities to facilitate the referral of samples to land-based reference laboratories.

For future expedition cruises, the objective should not be to transform ships into full reference laboratories. Rather, vessels travelling through high-risk ecological corridors should implement a tiered diagnostic preparedness strategy. This strategy could include basic point-of-care testing, syndromic clinical algorithms, telemedicine support from infectious disease specialists, sample collection kits, cold-chain procedures, and access to portable molecular platforms when feasible (33–35). The ship physician should not function as the sole diagnostic gatekeeper. A robust preparedness model should integrate the ship medical team with port health authorities, national public health agencies, reference laboratories, telemedicine networks, and international reporting mechanisms. This is particularly important for pathogens such as Andes orthohantavirus, where delayed recognition can affect case isolation, contact tracing, evacuation decisions, quarantine recommendations, and cross-border public health coordination. Table 3 outlines a proposed diagnostic preparedness package for expedition cruises operating in remote or zoonosis-prone settings.

**Table 3:** Proposed diagnostic preparedness package for expedition cruises.

Diagnostic level	Recommended capacity	Purpose
Clinical screening	Daily syndromic surveillance for fever, respiratory symptoms, and gastrointestinal symptoms	Support early identification of suspected cases
Basic point-of-care testing	Pulse oximetry, temperature, blood pressure, glucose, urine testing, rapid malaria or dengue tests where relevant, and basic inflammatory markers if available	Assess severity and support the initial differential diagnosis
Respiratory pathogen testing	Rapid antigen or molecular testing for common respiratory viruses	Identify common causes and guide isolation measures
Hantavirus-specific pathway	Pre-arranged access to RT-PCR or serology through reference laboratories; onboard sample collection kits	Confirm or rule out high-consequence zoonotic infections
Sample logistics	Swabs, blood tubes, cold-chain materials, and packaging compliant with infectious-substance transport requirements	Preserve diagnostic quality during evacuation or shipment
Telemedicine	24/7 access to infectious disease, intensive care, and public health experts	Support clinical and operational decision-making before evacuation
Reporting	Immediate notification pathways to port health authorities and national focal points	Enable rapid, IHR-compatible public health response
Training	Crew and medical staff training in PPE, sampling, isolation, documentation, and reporting	Reduce occupational risk and improve surveillance quality

IHR, International Health Regulations; PPE, personal protective equipment; RT-PCR, reverse-transcription polymerase chain reaction.

## 6 Governance and operational lessons from the MV *Hondius* outbreak

The MV *Hondius* outbreak should be interpreted not only as a zoonotic or shipboard event, but also as a stress test for international outbreak governance in expedition cruise settings. Existing instruments, including IHR 2005, WHO guidance for public health events on ships, and ECDC recommendations, provide a broad framework for notification, coordination, disembarkation, isolation, and contact tracing. However, the outbreak revealed that generic frameworks may be insufficient when a vessel with a high-consequence infectious disease is located far from tertiary care, involves passengers and crew from multiple countries, and requires urgent decisions about port access, evacuation, quarantine, repatriation, and post-travel monitoring.

Two operational gaps are particularly important. First, the reported uncertainty around safe port access and medical evacuation showed that expedition vessels may depend on *ad hoc* humanitarian, political, or operational decisions rather than pre-negotiated, binding procedures. Future preparedness should therefore include designated safe-port agreements for expedition itineraries, predefined medical evacuation corridors, receiving hospital arrangements, and escalation pathways involving the flag state, port state, operator, WHO, ECDC or regional public health bodies, and national IHR focal points. Second, the dispersal of passengers before definitive diagnosis illustrates the need for standardized traceability systems. Passenger manifests alone are insufficient. Operators should maintain auditable cabin assignments, dining and seating records, excursion participation logs, close-contact declarations, medical encounters, and post-disembarkation contact details in a format that can be rapidly transferred to public health authorities when a high-consequence infection is suspected.

These lessons do not imply that the international response failed entirely. On the contrary, the eventual coordination of medical evacuation, isolation, laboratory confirmation, and post-travel monitoring demonstrates the value of existing global health mechanisms. However, the outbreak also shows that expedition cruise preparedness cannot rely solely on reactive coordination after diagnosis. It requires expedition-specific protocols that define responsible actors, legal triggers, minimum operational standards, and accountability mechanisms before the ship departs. Table 4 presents an operational accountability matrix intended to translate One Health preparedness into concrete responsibilities.

**Table 4:** Operational accountability matrix for One Health preparedness in expedition cruises.

Phase	Governance or operational gap	Lead accountable actor(s)	Legal or policy basis	Required action	Accountability mechanism
Pre-route planning	Itinerary includes zoonosis-endemic regions, remote islands, or under-surveilled ecosystems	Cruise operator; expedition medical director; flag-state authority	ISM-code section 8	Conduct route-specific risk assessment	Written risk assessment retained for audit and shared with relevant port-health authorities when required
Pre-departure preparedness	No pre-arranged plan for safe port access, evacuation, receiving hospitals, or laboratory support	Cruise operator; flag state; port-state health authorities	IHR (2005) Articles 19,20, 22(h)	Pre-arrange safe-port, evacuation, laboratory, and telemedicine pathways	Signed operational protocol or memorandum of understanding for high-risk routes
Pre-boarding assessment	If the probable index case acquired the infection before embarkation and was still within the incubation period at the time of boarding, symptom-based screening alone would not have detected the risk	Cruise operator; ship medical officer; travel medicine provider	IHR (2005) Articles 23, 24	Training and strengthening onboard epidemiological surveillance, the early and timely detection of symptoms compatible with infection, the establishment of protocols for the isolation of suspected cases, and the implementation of infection prevention and control measures.	Auditable pre-boarding forms and predefined triggers for medical review
Onboard surveillance	Delayed recognition of nonspecific febrile illness	Ship medical officer; ship master	ISM-code section 8 IHR (2005) Article, 5 24(c)	Implement daily syndromic surveillance. Establish exercise and drill programmes to ensure preparedness for public health emergencies. Maintain conveyances free from sources of infection or contamination, including vectors and reservoirs, and implement appropriate measures to control sources of infection or contamination.	Daily medical log reviewed by the ship master and reportable to port-health authorities
Suspected high-consequence infection	Unclear triggers for isolation, escalation, and notification	Ship medical officer; ship master; operator medical lead	IHR (2005) Article 27	Isolate, protect staff, escalate clinically, and notify authorities	Time-stamped incident report and notification to the next port-health authority or national IHR focal point
Diagnostic escalation	Limited onboard capacity for rare zoonotic infections	Cruise operator; reference laboratory; receiving health authority	IHR (2005) Annex 1	Maintain sample, cold-chain, and reference-laboratory pathways	Documented chain of custody and target turnaround times for high-consequence pathogens
Safe port access and medical evacuation	Reliance on ad hoc port-access or humanitarian decisions	Port state; flag state; cruise operator; WHO or regional public health bodies	No specific binding legal provision currently identified	Pre-identify disembarkation sites, evacuation corridors, and receiving hospitals	Formal after-action review when port access is delayed, denied, or escalated
Passenger traceability	Passengers may disperse before definitive diagnosis	Cruise operator; public health authorities; national IHR focal points	IHR (2005) Article 23	Maintain auditable passenger, contact, excursion, and travel records	Transfer a standardized traceability package to authorities within 24 h of suspected high-consequence outbreak
Repatriation	Multi-country follow-up may become fragmented	National public health authorities; embassies or consulates; cruise operator	IHR coordination; national isolation or quarantine laws	Apply risk-stratified repatriation, isolation, and monitoring	Named responsible authority for each passenger or crew member until monitoring is completed
Post-travel monitoring	Long incubation periods may delay case detection	Receiving national health authority	Disease-specific WHO, ECDC, or CDC guidance	Monitor exposed contacts for the recommended incubation period	Centralized monitoring database and completion report
Risk communication	Conflicting messages may cause panic, stigma, or minimization	Public health authority; cruise operator; WHO or regional partners	IHR (2005) Article 6	Notify, through the most efficient means of communication available and via the National IHR Focal Point, within 24 h of the assessment of public health information, all events occurring within their territory that may constitute a Public Health Emergency of International Concern. In addition, the Regulations specify that, following notification, the State Party shall continue to communicate to the WHO all available public health information regarding the notified event, including any difficulties encountered and the support required to respond to the potential public health emergency.	-Documented communication coordination plan. -Designated lead communication authority. -Log of inter-state communications and message approvals. -Post-event communication review and lessons-learned report

IHR, International Health Regulations; WHO, World Health Organization; ECDC, European Centre for Disease Prevention and Control; CDC, Centers for Disease Control and Prevention.

## 7 Discussion

The MV *Hondius* outbreak is best interpreted as a sentinel event for expedition-era travel medicine and as a practical test of whether existing international preparedness mechanisms are sufficiently operational for remote, mobile, and multi-jurisdictional tourism settings (36–38). Unlike conventional mass tourism, expedition tourism intentionally brings travelers into biologically rich and often under-surveilled environments. These journeys may support science, conservation, education, and local economies, but they also create interfaces where ecological exposure, enclosed travel, delayed diagnosis, and international passenger dispersal can converge (39, 40).

The most important lesson is that the ship was not necessarily the ecological source of infection. Available evidence suggests that the probable index case may have acquired infection before boarding, during prior travel in the Southern Cone. In this context, the vessel functioned less as a reservoir and more as an amplifier of operational complexity. Once nonspecific symptoms occurred onboard, the response required clinical recognition, isolation, evacuation, laboratory confirmation, contact tracing, risk communication, and cross-border monitoring. Therefore, the key preparedness question is not only how to reduce ecological exposure before travel, but how to detect, contain, and coordinate response to an incubating or symptomatic infection after departure (8, 41, 42). Table 5 translates the main epidemiological and governance features of the MV *Hondius* outbreak into specific lessons and policy implications.

**Table 5:** Event-specific lessons learned from the MV *Hondius* outbreak.

Event feature	Gap revealed	Operational lesson	Policy implication
Probable infection before embarkation	Symptom-based pre-boarding screening may miss incubating infections	Exposure-history assessment is necessary but insufficient alone	Combine pre-boarding exposure assessment with onboard surveillance and post-travel monitoring
Nonspecific early symptoms	Febrile or gastrointestinal illness may not be recognized as a high-consequence zoonosis	Ship medical teams need differential diagnosis algorithms for severe travel-associated infections	Develop expedition-specific clinical escalation protocols for febrile illness in zoonosis-endemic routes
Andes virus identified after international laboratory work	Limited onboard diagnostic capacity delayed confirmation	Ships do not need full reference laboratories, but they need diagnostic escalation pathways	Require sample kits, cold-chain logistics, telemedicine access, and reference-laboratory agreements
Enclosed shipboard environment	Close and prolonged contact may increase risk for pathogens with limited human-to-human transmission	Early isolation and contact classification are critical	Define isolation triggers and close-contact criteria before departure
Remote itinerary and need for evacuation	Medical care depended on complex coordination across jurisdictions	Evacuation cannot be improvised during a high-consequence outbreak	Establish pre-negotiated safe-port and medical evacuation agreements for high-risk routes
Port-access uncertainty	Existing guidance may not guarantee operationally clear disembarkation pathways	Port-state, flag-state, operator, and international roles must be predefined	Create binding expedition-specific port-of-call outbreak protocols
Passenger dispersal before definitive diagnosis	Contact tracing becomes difficult after disembarkation and repatriation	Passenger manifests alone are insufficient	Require auditable cabin, dining, seating, excursion, medical, and post-disembarkation contact records
Multi-country passenger and crew composition	Follow-up may become fragmented across national systems	Each exposed person needs a named public health authority responsible for monitoring	Use IHR-compatible cross-border traceability packages and monitoring handover forms
Long incubation period	Cases may appear weeks after exposure or disembarkation	Surveillance must extend beyond the voyage	Implement risk-stratified monitoring for the full recommended incubation period
Public attention and media speculation	Naming individuals or speculating about unconfirmed sources may increase stigma	Epidemiological reporting should protect privacy and distinguish hypotheses from confirmed evidence	Avoid naming cases and clearly label unconfirmed exposure hypotheses

A purely reactive approach is insufficient. Once a symptomatic passenger has deteriorated onboard, disembarked, boarded a flight, or been hospitalized in another continent, the response becomes exponentially more complex. The appropriate model is anticipatory but also accountable: risks should be identified before travel, but responsibilities should also be assigned before travel. This includes identifying who authorizes medical evacuation, who receives the vessel, who triggers international notification, who receives traceability data, who monitors passengers after repatriation, and who conducts the after-action review.

The central lesson is not that birdwatching, cruises, island hopping, or ecological tourism are dangerous in themselves. The lesson is that their public health architecture has not evolved at the same speed as their geographic reach. Expedition cruises should no longer be managed only as tourism products or as generic ships under broad maritime health guidance. They should be treated as mobile One Health platforms requiring integrated planning across human health, animal health, environmental health, maritime medicine, port health, and international surveillance. This planning must be operational, auditable, and linked to clear authority and accountability.

## 8 Policy and practice recommendations

Expedition cruise preparedness should move from general guidance to route-specific, operationally accountable planning. Before itineraries involving orthohantavirus-endemic, zoonotic disease-endemic, or ecologically sensitive regions are approved, cruise operators, expedition medical teams, port authorities, and relevant public health agencies should conduct formal One Health risk assessments that include ecological exposure sites, rodent or wildlife interfaces, waste areas, rustic cabins, docks, food-storage sites, local medical capacity, diagnostic access, and evacuation feasibility. Passengers, guides, and crew should receive targeted pre-travel education on rodent and wildlife exposure, poorly ventilated structures, waste sites, food storage, and early symptoms requiring immediate medical reporting (43, 44).

Pre-boarding screening should not be considered sufficient for long-incubation zoonoses, particularly when exposure may have occurred before embarkation. Instead, expedition vessels should combine exposure-history assessment with onboard syndromic surveillance, clear isolation triggers, telemedicine support, and diagnostic escalation pathways. Ship medical teams should have access to preparedness packages that include sample collection materials, rapid tests, cold-chain logistics, and, where feasible, portable PCR or multiplex molecular platforms. BioFire-type syndromic PCR systems or equivalent tools may help identify common respiratory and gastrointestinal pathogens rapidly, while reference-laboratory pathways remain essential for hantavirus confirmation (45–47).

The MV *Hondius* outbreak also highlights the need for pre-agreed safe-port access, medical evacuation, and passenger traceability protocols. Port authorities and cruise operators should establish outbreak response pathways before departure, including designated disembarkation points, receiving hospitals, isolation capacity, laboratory referral routes, and mechanisms for rapid transfer of passenger manifests, cabin assignments, seating charts, excursion logs, medical records, and post-disembarkation contact details. Responsible health authorities should ensure timely case investigation, risk assessment, intersectoral coordination, contact tracing, and post-travel monitoring to prevent secondary transmission and wider international spread. Public communication should remain factual, transparent, proportionate, and privacy-preserving, avoiding both panic and minimization (48, 49).

## 9 Conclusion

The MV *Hondius* outbreak demonstrates how a probable ecological exposure can evolve into a multi-country public health event when it intersects with expedition tourism, enclosed shipboard environments, delayed diagnostic recognition, medical evacuation, and international repatriation. The main lesson is not simply that expedition cruises require greater awareness of zoonotic risk, but that existing global health guidance must be translated into binding, expedition-specific, and auditable protocols. Safe port access, medical evacuation, onboard surveillance, diagnostic escalation, passenger traceability, and post-travel monitoring cannot be improvised after a high-consequence infection is suspected. They must be planned before departure, assigned to responsible actors, and evaluated after the event. Expedition cruises, island hopping, birdwatching, and remote ecological tourism are not inherently unsafe; however, their expanding geographic reach requires a preparedness model that integrates environmental risk assessment with operational accountability. In an era of high-mobility ecological tourism, expedition vessels should be viewed not only as transport platforms, but as mobile sentinels within the global One Health system.

## Data Availability

The original contributions presented in the study are included in the article/supplementary material, further inquiries can be directed to the corresponding author.
